# Comparison of Modified Asymmetric Inverse Z-plasty and Z-plasty in the Correction of Epicanthal Folds

**DOI:** 10.1007/s00266-025-05282-0

**Published:** 2025-10-20

**Authors:** Zhiqiang He, Weinan Zhang, Xi Yu, Hao Qin, Junfei Teng, Jinqing Xie

**Affiliations:** 1 Department of Plastic Surgery, Chongqing Dangdai Plastic Surgery Hospital, Chongqing, 400020 China; 2 Department of Dermatology of Jiangbei Campus (The 958th Hospital of Chinese People’s Liberation Army), The First Affiliated Hospital of Army Medical University, Chongqing, 400020 China; 3 Center of urology and nephrology, The Second Affiliated Hospital of Chongqing Medical University, Chongqing, 401336 China

**Keywords:** Epicanthoplasty, Modified asymmetric inverse Z-plasty, Z-plasty, Scar, Patient satisfaction

## Abstract

**Background:**

Epicanthoplasty is the most common cosmetic eye surgery. However, traditional surgical strategies usually cause visible scars in the medial canthus. This study innovatively aimed to propose a modified asymmetric inverse Z-plasty derived from the traditional Z-plasty technique to minimize postoperative scars and consequently enhance patient satisfaction.

**Methods:**

This retrospective study included 172 patients with epicanthal folds who underwent an epicanthoplasty. Group A patients (*n* = 94) underwent modified asymmetric inverse Z-plasty, whereas Group B patients (*n* = 78) underwent Z-plasty. Postoperative outcomes, including scarring (assessed using the Vancouver scar scale [VSS]), scar hiding degree, patient satisfaction, and recurrence rates, were evaluated at 6 months.

**Results:**

Postoperative evaluations revealed that both groups achieved normal wound healing without infections or complications after 7 days. VSS assessments indicated superior scar outcomes in Group A patients with moderate-to-severe epicanthal folds compared with those in Group B participants. Scar hiding degree analysis demonstrated a significantly higher percentage of postoperative pure hidden scars in Group A (*P* = 0.0042), particularly in moderate (*P* < 0.0001) and severe epicanthal folds (*P* = 0.002). Patient satisfaction was significantly higher in Group A (79.8%, very satisfied) than in Group B (47.4%, *P* < 0.0001). However, no significant difference in recurrence rates was observed between the two groups.

**Conclusion:**

Patients treated with modified asymmetric inverse Z-plasty exhibited superior scar hiding degree and overall satisfaction compared to those who underwent Z-plasty. Hence, modified asymmetric inverse Z-plasty is an effective treatment for patients with epicanthal folds, particularly in moderate-to-severe cases.

**Level of Evidence IV:**

This journal requires that authors assign a level of evidence to each article. For a full description of these Evidence-Based Medicine ratings, please refer to the Table of Contents or the online Instructions to Authors www.springer.com/00266.

## Important Points

1. The modified asymmetric inverse Z-plasty improved the insufficient longitudinal skin at the inner canthus by incorporating an upper eyelid rotation flap, thereby theoretically reducing incision tension and scar formation.
2. Compared with Z-plasty, the modified asymmetric inverse Z-plasty was more effective in hiding scars, especially in patients with moderate-to-severe epicanthus.
3. Patients who underwent the modified asymmetric inverse Z-plasty for epicanthus correction exhibited higher overall satisfaction than those treated with Z-plasty.
4. By improving the rotation angle and range of the flap, the modified asymmetric inverse Z-plasty prevented re-adhesion of released tissue to its original position and reduced the risk of epicanthus recurrence.

## Introduction

Epicanthal folds are obliquely or vertically distributed flap-like skin covering the anterior zone of the medial canthus angle. It covers the lacrimal caruncle of the medial canthus, making the eye appear shorter and less aesthetically pleasing. Severe cases are usually accompanied by ptosis, blepharophimosis, or other deformities. Owing to thicker eyelid skin and lower nose bridge in Asians [1], the incidence of epicanthal folds is higher in Asians (approximately 40%) than in non-Asians (< 5%) [2, 3]. According to the formation characteristics of the epicanthal folds in East Asians, epicanthoplasty can obviously increase the width of the palpebral fissure, reduce the spacing between the medial canthi, and increases the visual aesthetic effect on the eyes and face. Therefore, epicanthoplasty has become a significant oculoplastic surgery among Asians [4].

Previous studies have reported excess transverse skin as the primary cause of epicanthal folds; hence, transverse skin excision is used to correct the condition [5, 6]. However, owing to the high tension at the incision site, scars are easily formed in such surgical procedures, causing postoperative cicatricial epicanthal folds. Notably, further research has indicated that in addition to excess transverse skin, epicanthal folds are caused by insufficient longitudinal skin [6, 7]. Therefore, surgeons proposed to improve the uneven skin distribution of the medial canthus using various flap techniques, such as Z-plasty [8], Park Z-plasty [9], “Y-V”-plasty [10], Mustarde’s four-flap method [11], and skin redraping [12]. These procedures alter the direction of skin tension in the medial canthal region and release deep structures, resulting in excellent therapeutic outcomes. Nevertheless, they induce postoperative complications, including scarring, medial canthus asymmetry, and insufficient correction. Some patients develop significant cicatricial hyperplasia, which negatively affects their appearance [13, 14]. Hence, novel surgical techniques for correcting epicanthal folds should be explored.

Since 2015, we have investigated a modified asymmetric inverse Z-plasty, an adaptation of the traditional Z-plasty technique for correcting epicanthal folds, to achieve superior therapeutic outcomes. This retrospective study aims to compare postoperative scarring, overall patient satisfaction, and epicanthal fold recurrence between Z-plasty and modified asymmetric inverse Z-plasty in treating epicanthal folds to provide a better treatment option for correcting epicanthal folds and significantly enhancing postoperative aesthetic outcomes.

## Patients and Methods

### Patient Population

This was a retrospective study. A total of 256 patients who underwent epicanthoplasty between January 2022 and October 2023 under the author’s supervision were randomly selected for objective evaluation. The final cohort of enrolled patients was determined following a rigorous screening process based on the predefined inclusion and exclusion criteria. The inclusion criteria were as follows: (1) healthy physical and mental states, a strong desire for surgery, and no contraindications; (2) presence of varying degrees of epicanthal folds; (3) taking of photographs preoperatively and at 7 days and 6 months postoperatively. The exclusion criteria were as follows: (1) patients who could not be followed up for more than 6 months after surgery; (2) patients who received photoelectric cosmetology, injection, or other cosmetic treatments after surgery; (3) patients with other systemic or local diseases that affected incision healing or medial canthus morphology; (4) patients with unsuccessful epicanthoplasty; (5) patients with cicatricial diathesis. After screening based on the inclusion and exclusion criteria, 172 patients participated in scar assessment and comparison.

**Fig. 1:**
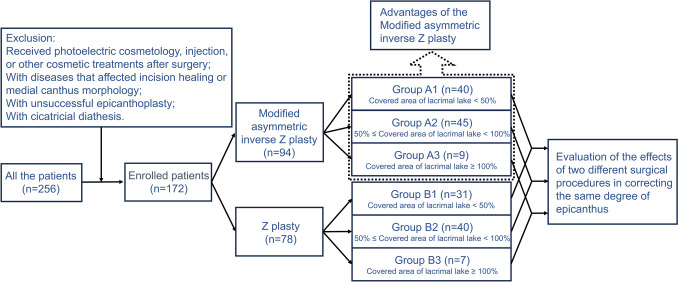
Scheme for patients’ distribution

Patients with epicanthal folds were classified into two groups: those who underwent modified asymmetric inverse Z-plasty combined with double eyelid plasty (Group A) and those who underwent Z-plasty combined with double eyelid plasty (Group B) (Fig. 1). According to the severity of the epicanthal folds [15], groups A and B were divided into three subgroups: A1, A2, and A3; B1, B2, and B3 (Fig. 1).

### Preoperative Design

All patients were instructed to remain in the supine position. In Group A, the medial canthus skin was pulled toward the nasal side to expose the medial canthus. The skin was then released, and a new point (A) was marked on the vertical projection of the skin at the medial canthus. Based on the patient’s preferences and aesthetic design, Point A could also be appropriately adjusted inward to the nasal side by 1–2 mm. Point B was taken as the endpoint of the free margin of the epicanthal folds at the lower eyelid, and line AB was marked. The epicanthal fold margin was marked from Point B to the starting point of the upper eyelid (Point C). The epicanthal folds were pulled to the nasal side to expose the medial canthus, followed by the medial canthus skin from Point C to the endpoint which was marked as Point D. Line CD (the short arm) was parallel to Line AB (the long arm). Occasionally, it was necessary to adjust the length of Line AB in advance based on the coverage range of the medial canthus by the folds and the symmetry of the medial canthus on both sides. In Group B, a new medial canthus point A was first set, representing the projection point of the innermost end of the medial canthus on the skin. The starting point of the epicanthal fold of the upper eyelid and the endpoint of the free margin of the lower eyelid were marked as B and C, respectively. Point D was marked after pulling the epicanthal folds to the nasal side. The lengths of AB, BC, and CD were made to be equal to enable the suturing of BC to CD and the suturing of transposed BC to AB, ensuring no discrepancy in the length between opposing skin margins at the suture sites. Two triangular flaps of the same size were formed in ABC and BCD areas. In addition, a narrow area of thin eyelid skin existed between Point B and the thick nasal skin at Point A. Thus, Line AB was confined to this thin skin to avoid hypertrophic scarring, as incisions on the thick nasal skin may cause thick scars [16].

### Surgical Procedures

**Fig. 2:**
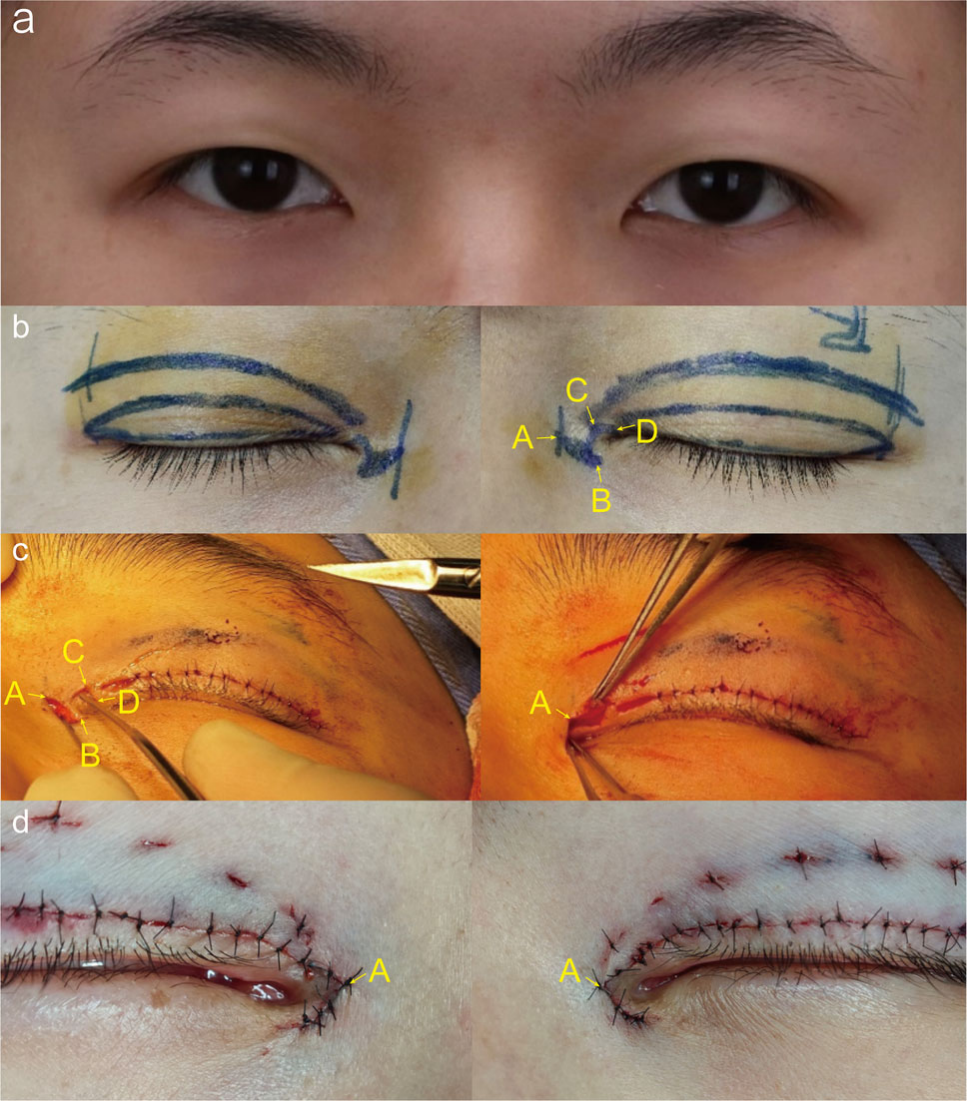
Preoperative design and specific procedure of modified asymmetric inverse Z-plasty. **a** Preoperative frontal view. **b** The design of incisions. **c** Sequentially incised AB and CD along the marked line. The flap between BC was trimmed into an arc shape, the flap was rotated, and the medial canthus point was repositioned. The upper eyelid margin flap was cut and trimmed to fully relieve tension. **d** The immediate view of the medial canthus after the operation

*Group A*: The surgical area was disinfected with povidone–iodine and covered with sterile towels, followed by the administration of local anesthesia (2% lidocaine + 1:100,000 adrenaline). Subsequently, AB and CD were cut along the marked line using a #11 scalpel blade. Surgical scissors were used to cut the epicanthal folds at Points B and C toward the center for the arc formed by the BC line to resemble a normal medial canthus. Next, surgical scissors were used to fully separate the subcutaneous layer of the medial canthus area. The adhesion between the skin and some muscle layers was released, a muscle flap was formed with a thin layer of the orbicularis oculi muscle, and the white fibrous adhesive tissue on the surface of the medial canthus muscle layer was removed. After full release, the “cat ear” skin at the upper margin of Line AB was trimmed, and the midpoint of Line BC was sutured to Point A using a 7-0 nylon thread to form a new medial canthus point. A 5-mm incision was made at the outer margin of Point D near the upper eyelid margin (1 mm near the eyelash root), and the surgical scissors were used to cut open the skin from the incision to Point D along the upper eyelid margin. The skin and subcutaneous orbicularis oculi muscle were fully released, the excess orbicularis oculi muscle and skin were trimmed, and the incisions were closed with a 7-0 nylon thread using tension-free interrupted suturing (Fig. 2).

**Fig. 3:**
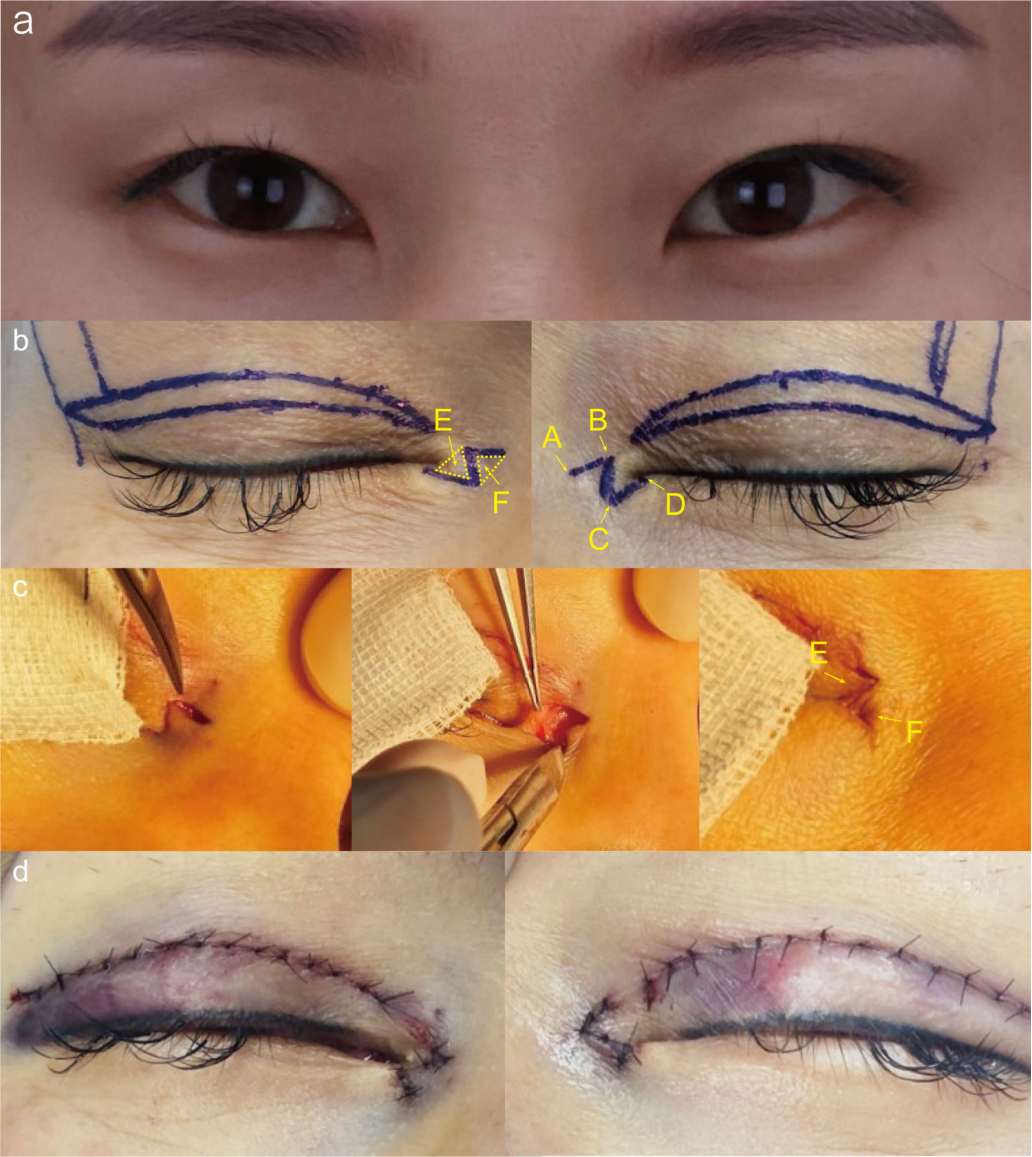
Preoperative design and specific procedure of Z-plasty. **a** Preoperative frontal view. **b** The design of incisions. **c** AB, BC, and CD were sequentially incised along the marked line, the flap was fully released and rotated, and the triangular flaps E and F were transposed and sutured. **d** The immediate view of the medial canthus after the operation

*Group B*: After disinfection and anesthesia, the skin was incised along the designed points A, B, C, and D. Surgical scissors were then used to release the adhesions between the skin and muscle layers. The ABC flap was dissected and transposed to the BCD area, with point C sutured to point A and point B to point D, and the incision was sutured in the same way as in Group A (Fig. 3).

**Fig. 4:**
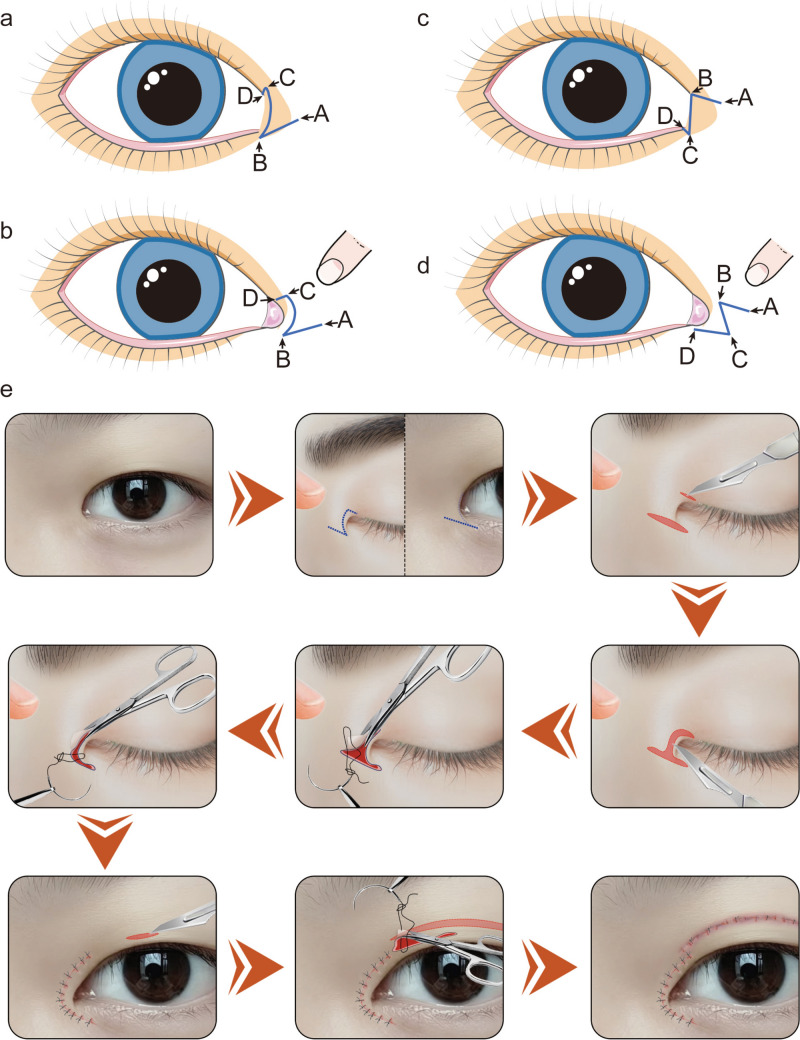
Schematic diagrams of the surgical designs of modified asymmetric inverse Z-plasty and Z-plasty. **a** Location of incision design line for modified asymmetric inverse Z-plasty in the natural state. **b** Location of incision design line for modified asymmetric inverse Z-plasty after exposing the lacrimal caruncle. **c** Location of incision design line for Z-plasty in the natural state. **d** Location of incision design line for Z-plasty after exposure of the lacrimal caruncle. **e** The step-by-step procedure for modified asymmetric inverse Z-plasty

To better demonstrate the differences between the two surgical designs, we created simplified schematic diagrams of the two surgical procedures. The positions of the incision design lines of the modified asymmetric inverse Z-plasty in the natural state and after exposure of the lacrimal caruncle are shown in Fig. 4a, b, respectively. The incision design line positions of Z-plasty in the natural state and after exposure of the lacrimal caruncle are shown in Fig. 4c, d, respectively. To demonstrate our surgical procedure clearly, we created a simplified schematic diagram of the modified asymmetric inverse Z-plasty (Fig. 4e).

### Evaluation Indicators

Subjective and objective evaluations were conducted. The subjective evaluation involved assessing the overall satisfaction of patients with a scar on the medial canthus 6 months post-surgery. The objective evaluation involved taking photos and observing the wound healing of the patients 7 days post-surgery, as well as taking photos to evaluate the scar hiding degree at the medial canthus and epicanthal fold recurrence 6 months post-surgery.

We used a blinded assessment approach to analyze scar characteristics. All surgical procedures were performed uniformly by the same senior plastic surgeon to ensure standardized epicanthoplasty. Image data collection adhered to a standardized protocol. During the preoperative baseline phase and the 6-month postoperative follow-up, a Canon R10 SLR camera (fitted with an EF 50 mm f/1.8 STM fixed-focus lens) mounted on a tripod was used to capture multi-angle photographs of all participants at a consistent shooting distance of 1 m within a controlled photographic environment. The assessment stage involved a rigorous double-blind design, and all digital images were anonymized through an unlabeled presentation and assigned random numbers. Scar characteristics were evaluated by two plastic surgeons, each with more than 10 years of clinical experience, in an independent and blinded setting. The evaluators did not participate in the surgical procedures and were not provided with the clinical information before the assessment.

1. Wound healing [17]: The incisions were evaluated for closure, redness, no evident swelling, effusion, blood exudation, and reduced pain.
2. Scar assessment was conducted using the Vancouver Scar Scale (VSS) [18, 19], which quantifies the overall scar severity by evaluating four clinical parameters: pigmentation, pliability, vascularity, and height (Table 1). All scars were independently evaluated by two senior plastic surgeons blinded to the treatment groups, and the average of their assessments was recorded.
3. Degree of scar hiding [4]: a. Hidden: Scar located in the medial canthus but not visible; b. Partially hidden: Some scars visible outside the medial canthus; c. No hidden: Scar fully exposed outside the medial canthus and visible.
4. Patient satisfaction was rated as very satisfied, satisfied, somewhat satisfied, or dissatisfied.
5. Epicanthal fold recurrence: a. No recurrence: lacrimal caruncle fully exposed without epicanthal folds recurrence; b. Mild recurrence: Epicanthal folds retraced < 1/3 of the pre-surgery size; c. Significant recurrence: Epicanthal folds retraced ≥ 1/3 of the pre-surgery size.

**Table 1:** Vancouver Scar Scale (VSS)

Variables	Contents	Score
Pigmentation (0–2)	Normal	0
	Hypopigmentation	1
	Hyperpigmentation	2
Vascularity (0–3)	Normal	0
	Pink	1
	Red	2
	Purple	3
Pliability (0–5)	Normal	0
	Supple	1
	Yielding	2
	Firm	3
	Banding	4
	Contracture	5
Height (0–3)	Normal (flat)	0
	0–2 mm	1
	2–5 mm	2
	> 5 mm	3

### Data Analysis

All statistical analyses were conducted using SPSS software (version 25.0 and 29.0, IBM SPSS Statistics). Continuous variables were presented as mean ± standard deviation (mean ± SD). Unpaired *t*-tests were used to compare continuous variables. Chi-squared tests were used to compare categorical variables. Statistical significance was set at *P* < 0.05.

## Results

### Patient Data

Following the application of the inclusion and exclusion criteria, the study comprised 172 patients, including 94 with epicanthal folds who underwent modified asymmetric inverse Z-plasty combined with double eyelid plasty (Group A) and 78 with epicanthal folds who underwent Z-plasty combined with double eyelid plasty (controls, Group B). In Group A, 40 patients had mild epicanthal folds (Group A1, mean age: 28.94 ± 6.63 years), 45 had moderate epicanthal folds (Group A2, mean age: 29.18 ± 7.22 years), and 9 had severe epicanthal folds (Group A3, mean age: 27.11 ± 5.74 years) (Table 2). In Group B, 31 patients had mild epicanthal folds (Group B1, mean age: 28.94 ± 6.62 years), 40 had moderate epicanthal folds (Group B2, mean age: 28.90 ± 6.02 years), and 7 had severe epicanthal folds (Group B3, mean age: 26.71 ± 6.02 years) (Table 2). The differences in the ages of patients with different epicanthal fold grades between groups A and B were not statistically significant (*P* > 0.05). The medial canthus incisions in groups A and B healed normally without infection, abnormal wound healing, increased pain, or other abnormalities 7 days post-surgery.

**Table 2:** Demographic and baseline characteristics of the two groups

Characteristic value	Modified asymmetric inverse Z-plasty	Z-plasty	*P*-value
Sex			
Male	0	0	
Female	94	78	
Epicanthal fold grade			0.906
Mild	40	31	
Moderate	45	40	
Severe	9	7	
Age (years)			
Mean ± SD	28.99 ± 9.57	28.72 ± 8.39	
Mild^*^	28.94 ± 6.63	28.94 ± 6.62	0.95
Moderate^*^	29.18 ± 7.22	28.90 ± 6.02	0.913
Severe^*^	27.11 ± 5.74	26.71 ± 6.02	0.90

^*^The age of patients was divided into 3 groups according to epicanthal fold grade.

### VSS in Patients with Mild, Moderate, and Severe Epicanthal Folds

We assessed outcomes of the scar 6 months postoperatively using VSS. The results indicated that, in patients with mild epicanthal folds, the mean score for Group A was 2.30 ± 0.65, while that for Group B was 2.29 ± 0.67 (Table 3). No statistically significant differences were observed between the two groups (*P* = 0.925; Table 3). In patients with moderate epicanthal folds, the mean score for Group A was 3.69 ± 0.70, while that for Group B was 5.13 ± 0.69 (Table 3). Group A had a significantly lower score than did Group B (*P* < 0.0001; Table 3). For patients with severe epicanthal folds, the mean score for Group A was 5.22 ± 0.44, while that for Group B was 7.57 ± 0.54 (Table 3). The score of Group A was significantly lower than that of Group B (*P* < 0.0001; Table 3). These results indicate that patients with moderate-to-severe epicanthal folds who underwent modified asymmetric inverse Z-plasty had better scar outcomes than did those who underwent Z-plasty 6 months postoperatively.

**Table 3:** Scar assessment with VSS after epicanthoplasty

Epicanthal folds	Group A (*n* = 94) Mean (SD)	Group B (*n* = 78) Mean (SD)	*P*-value
Mild	2.30 (0.65)	2.29 (0.67)	0.925
Moderate	3.69 (0.70)	5.13(0.69)	<0.0001
Severe	5.22 (0.44)	7.57 (0.54)	<0.0001

### Postoperative Score of Scar Hiding Degree in Patients with Epicanthal Folds

Additionally, we assessed scar outcomes 6 months postoperatively using the scar hiding degree [4]. In Group A, the scar was pure hidden in 67 patients (71.3%), partially hidden in 27 (28.7%), and no hidden in 0 (Table 4). In Group B, the scars were pure hidden in 29 patients (37.2%), partially hidden in 31 (39.7%), and no hidden in 18 (23.1%) (Table 4). The difference in the overall degrees of postoperative scar hiding between groups A and B was statistically significant (*P* = 0.0042; Table 4). The pure hidden rate in the postoperative scar hiding degree was higher in patients who underwent modified asymmetric inverse Z-plasty than in those who underwent Z-plasty (71.3% vs. 37.2%), demonstrating that the modified asymmetric inverse Z-plasty exhibited a promising effect on hiding the scars.

**Table 4:** Comparison of postoperative medial canthus scar hiding degree of patients between groups A and B

	Pure hidden	Partially hidden	No hidden	*P*-value
Group A (*n* = 94)	67 (71.3%)	27 (28.7%)	0	0.0042
Group B (*n* = 78)	29 (37.2%)	31 (39.7%)	18 (23.1%)	

**Fig. 5:**
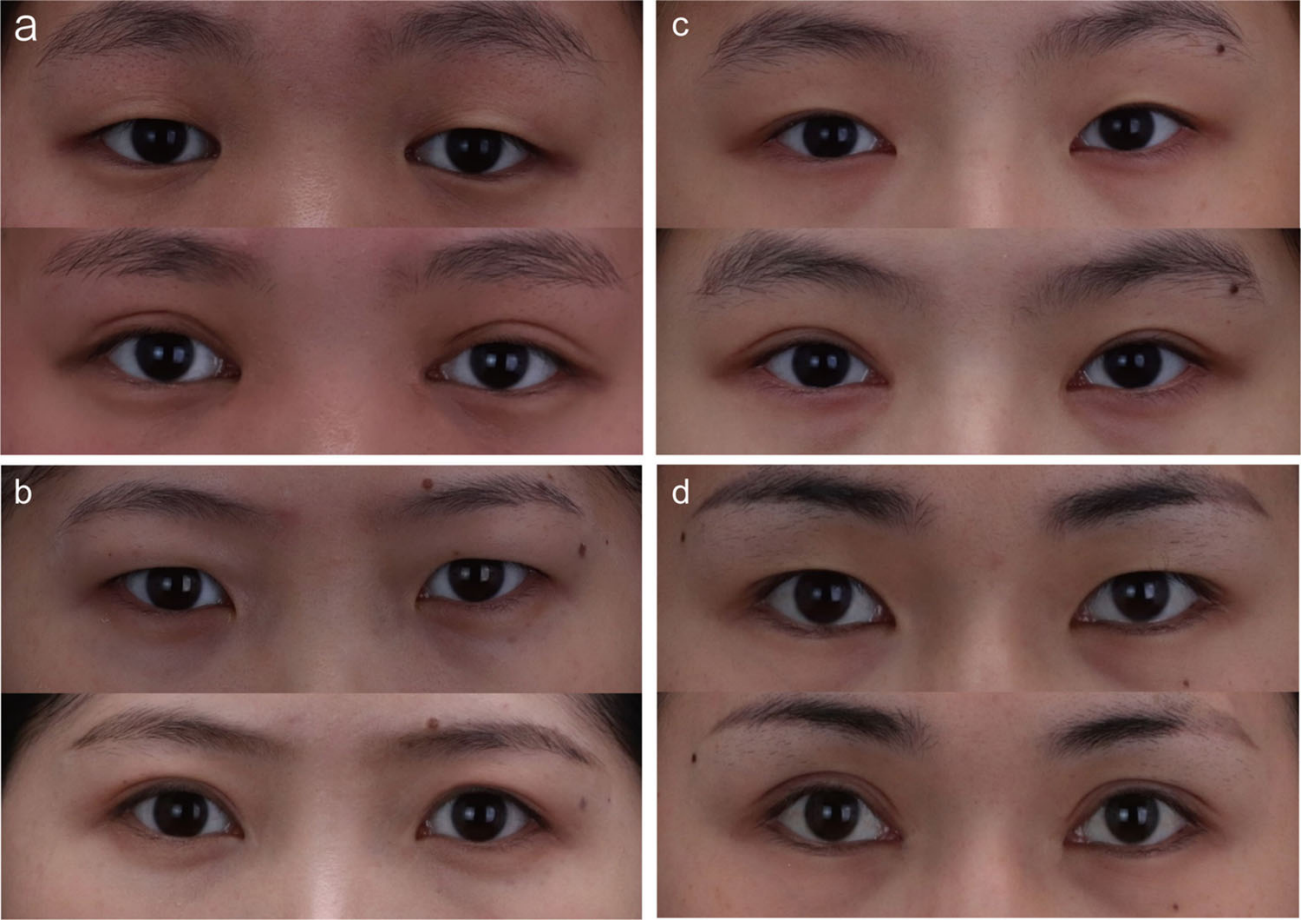
Pictures of patients with mild epicanthal folds. **a** and **b**. The pictures of patients with mild epicanthal folds before (top) and after (bottom) modified asymmetric inverse Z-plasty. **c** and **d**. The pictures of patients with mild epicanthal folds before (top) and after (bottom) Z-plasty. The red arrows indicate the scar

**Fig. 6:**
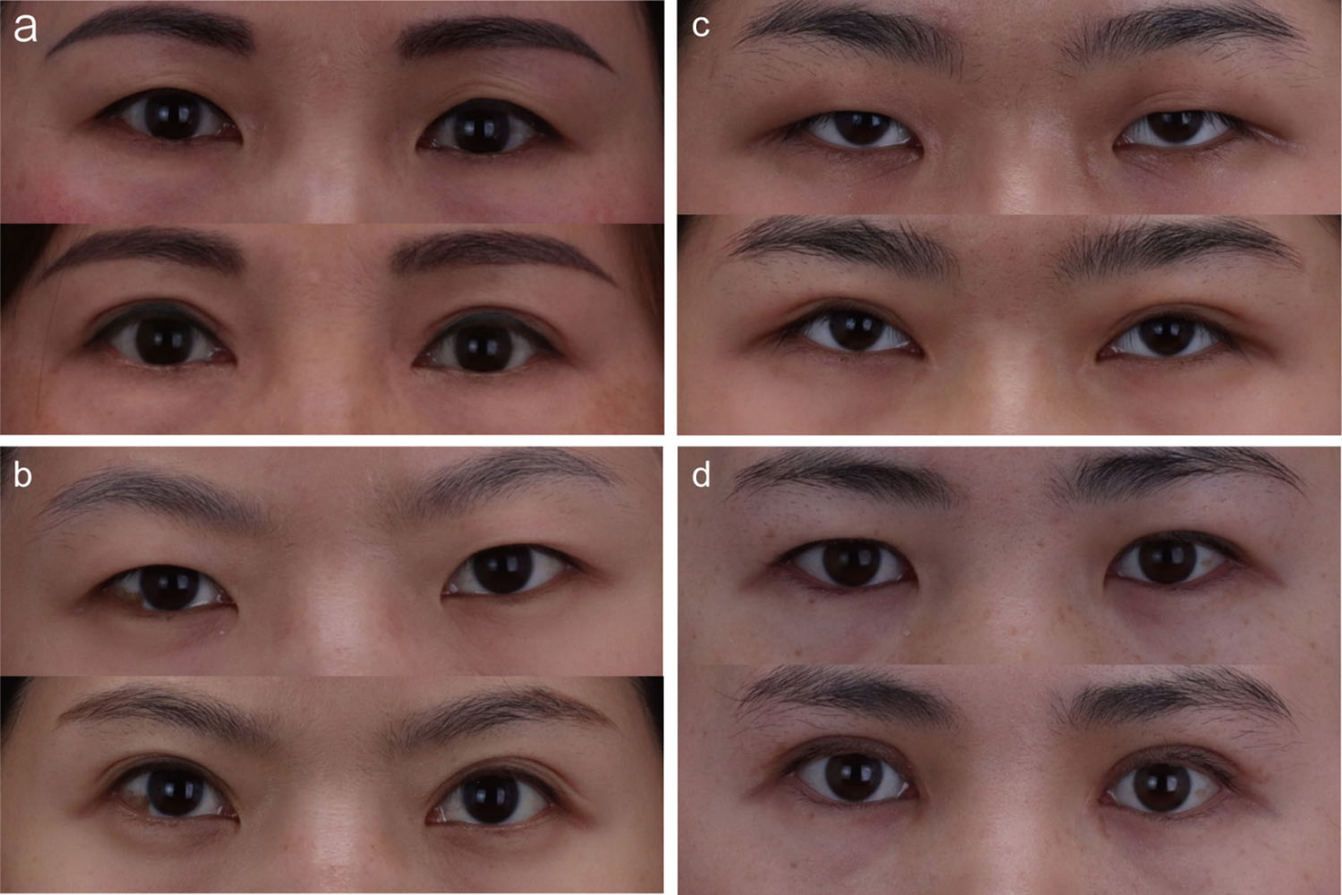
Pictures of patients with moderate epicanthal folds. **a** and** b**. The pictures of patients with moderate epicanthal folds before (top) and after (bottom) modified asymmetric inverse Z-plasty. **c** and **d**. The pictures of patients with moderate epicanthal folds before (top) and after (bottom) Z-plasty. The red arrows indicate the scar

**Fig. 7:**
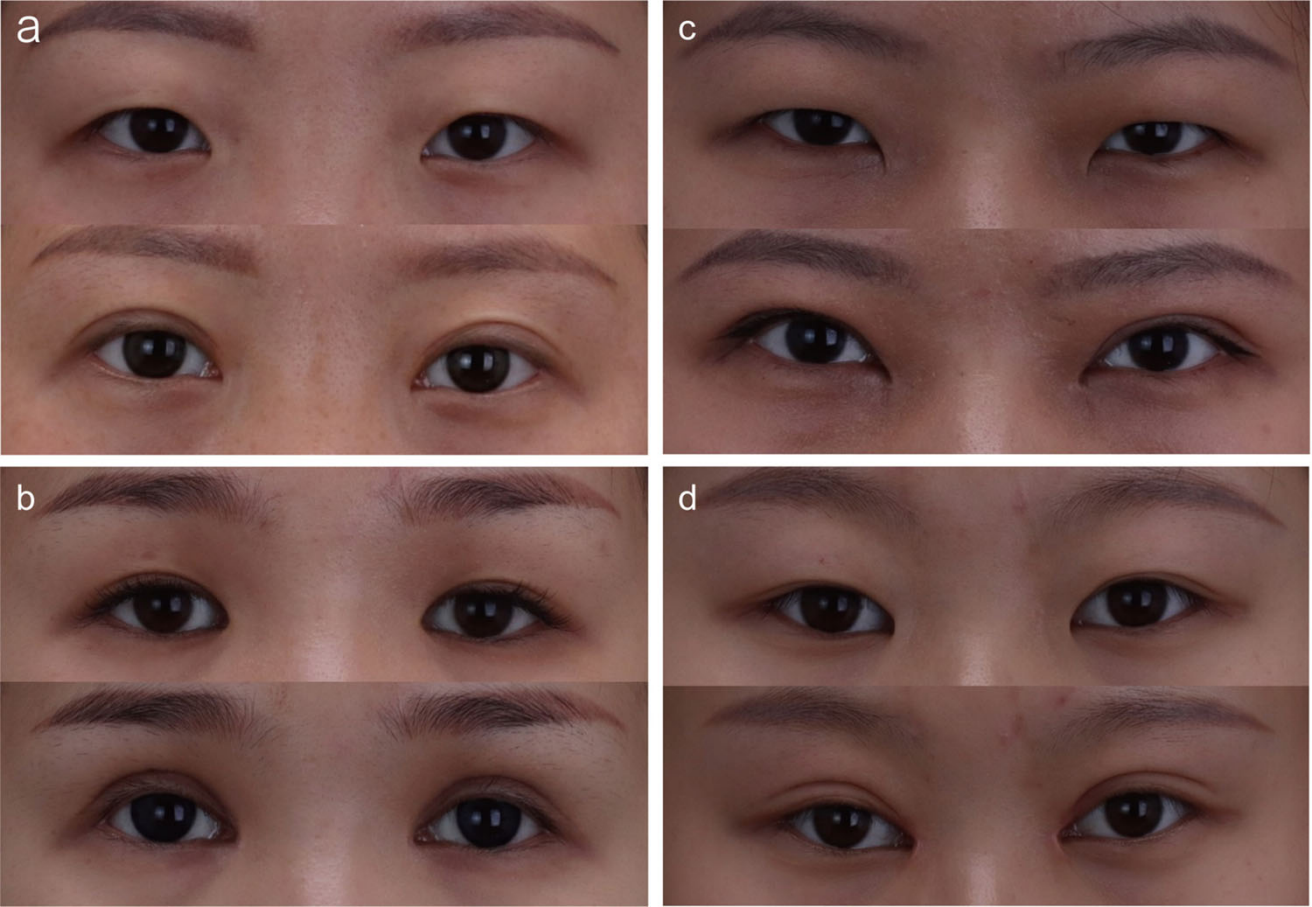
Pictures of patients with severe epicanthal folds. **a** and** b**. The pictures of patients with severe epicanthal folds before (top) and after (bottom) modified asymmetric inverse Z -plasty. **c** and **d** The pictures of patients with severe epicanthal folds before (top) and after (bottom) Z-plasty. The red arrows indicate the scar

Furthermore, we investigated whether patients with different degrees of epicanthal folds who underwent these two surgeries exhibited different degrees of postoperative scar hiding. In Group A1, there were 38 patients (95%) with pure hidden scars, 2 (5%) with partially hidden scars, and 0 with no hidden scars, whereas 25 patients (80.6%) had pure hidden scars, 6 (19.4%) had partially hidden scars, and 0 had no hidden scars in Group B1 (*P* = 0.0578) (Table 5). Notably, the differences were not statistically significant. There were 27 patients (60%) with pure hidden scars, 18 (40%) with partially hidden scars, and 0 with no hidden scars in Group A2; 4 patients (10%) with pure hidden scars, 24 (60%) with partially hidden scars, and 12 (30%) with no hidden scars in Group B2 (*P* < 0.0001) (Table 5). In addition, there were two patients (22.2%) with pure hidden scars, seven (77.8%) with partially hidden scars, and none with no hidden scars in Group A3; no patients had pure hidden scars, one (14.3%) had partially hidden scars, and six (85.7%) had no hidden scars in Group B3 (*P* = 0.002) (Table 5). The percentages of pure hidden scars in groups A2 and A3 were significantly higher than those in groups B2 and B3. These results suggest that the modified asymmetric inverse Z-plasty is more effective than Z-plasty in hiding scars in patients with moderate and severe epicanthal folds. The scars hidden in each group are shown in Figs. 5, 6, and 7.

**Table 5:** Comparison of scar hiding degree after correction of epicanthus folds of the same degree by two surgical procedures

	Pure hidden	Partially hidden	No hidden	*P*-value
Group A1 (*n* = 40)	38 (95%)	2 (5%)	0	0.0578
Group B1 (*n* = 31)	25 (80.6%)	6 (19.4%)	0	
Group A2 (*n* = 45)	27 (60%)	18 (40%)	0	<0.0001
Group B2 (*n* = 40)	4 (10%)	24 (60%)	12 (30%)	
Group A3 (*n* = 9)	2 (22.2%)	7 (77.8%)	0	0.002
Group B3 (*n* = 7)	0	1 (14.3%)	6 (85.7%)

### Evaluation of Patient Satisfaction

In Group A, 75 patients (79.8%) were very satisfied, 11 (11.7%) were satisfied, 8 (8.5%) were somewhat satisfied, and none were unsatisfied. In Group B, 37 patients (47.4%) were very satisfied, 21 (26.9%) were satisfied, 15 (19.3%) were somewhat satisfied, and 5 (6.4%) were unsatisfied (*P* < 0.0001) (Table 6). The proportion of very satisfied patients was higher in Group A than in Group B, indicating that modified asymmetric inverse Z-plasty was more effective than Z-plasty in correcting the epicanthal folds.

**Table 6:** Comparison of overall postoperative satisfaction of patients between groups A and B

	Very satisfied	Satisfied	Somewhat satisfied	Unsatisfied	*P*-value
Group A (*n* = 94)	75 (79.8%)	11 (11.7%)	8 (8.5%)	0	<0.0001
Group B (*n* = 78)	37 (47.4%)	21 (26.9%)	15 (19.3%)	5 (6.4%)	

### Evaluation of Recurrence of Epicanthal folds

We assessed the epicanthal fold recurrence. In Group A, 92 patients (97.9%) had no recurrence, 2 (2.1%) had a mild recurrence, and 0 had significant recurrence (Table 7). In Group B, 74 patients (94.9%) had no recurrence, 4 (5.1%) had a mild recurrence, and 0 had a significant recurrence (Table 7). There was no significant difference in recurrence rates between the two groups (*P* = 0.286; Table 7).

**Table 7:** Comparison of postoperative epicanthal fold recurrence between groups A and B

	No recurrence	Mild recurrence	Significant recurrence	*P*-value
Group A (*n* = 94)	92 (97.9%)	2 (2.1%)	0	0.286
Group B (*n* = 78)	74 (94.9%)	4 (5.1%)	0	

## Discussion

Epicanthal folds are skin folds that cover the inner corner of the eyes, potentially altering their shape and influencing their overall appearance. In more severe cases, the epicanthal folds may be associated with ptosis or other deformities. Asians exhibit a significantly higher incidence rate than do non-Asians, which may be attributed to their thicker eyelid skin and lower nose bridges. Epicanthoplasty effectively enlarges the palpebral fissure, appropriately shortens the interocular distance, and significantly enhances the aesthetic appearance of the eyes and face. Consequently, this procedure has become a significant and common plastic surgical technique in Asia. The skin at the medial canthus is the thinnest in the body, serves as the endpoint for multiple anatomical structures, and is subject to relatively high tension [20]. The anatomical abnormalities of the epicanthal folds include excess skin in the horizontal direction and insufficient skin in the vertical direction of the medial canthus, dislocation and misalignment of the deep and shallow head fibers of the orbicularis oculi muscle at the beginning of the medial canthus, and abnormal tension at the medial canthus skin caused by excess orbicularis oculi muscle and fibrous adipose tissue [21]. Common surgical techniques for epicanthal fold correction include Z-plasty, Y-V plasty, and skin redraping techniques. Despite their relatively favorable therapeutic outcomes in the correction of epicanthal folds, these methods have certain limitations. Z-plasty and Y-V-plasty only involve using the skin in the horizontal direction of the epicanthal folds to supplement the insufficient skin in the vertical direction. Owing to the limited skin in the overall area of the epicanthal folds, the local skin is relatively insufficient, even after vertical supplementation, resulting in tension at the incision and postoperative scar formation. Additionally, the skin redraping technique expands the medial canthus by fully releasing the subcutaneous tissue of the epicanthal folds and excising redundant transverse skin. However, this technique cannot address the challenge of insufficient vertical skin, and the lateral incision in the lower eyelid tends to be relatively long, thereby increasing the risk for scar formation. Therefore, developing novel surgical approaches is urgent to achieve superior therapeutic outcomes.

Based on the anatomical characteristics of the epicanthal folds, we modified inverse Z-plasty by designing two flaps of different lengths, termed modified asymmetric inverse Z-plasty. The surgical procedure involved cutting off the ectopic orbicularis oculi muscle, fully separating the subcutaneous tissue, breaking the fibrous connection between the skin and the muscle, completely releasing the tension of the facial expression muscles on the medial canthus, and relocating the new medial canthus point according to the preoperative design to promote healing during skin layering. Clinical and surgical practices have revealed that most patients with single eyelids have excess skin on the upper eyelid; therefore, we designed a rotational flap on the upper eyelid margin by taking advantage of the redundancy and malleability of the skin on the upper eyelid. This surgical procedure prevented the released tissue from re-adhering to the original position, and the vertical skin at the medial canthus was further supplemented by designing an eyelid margin incision flap to correct the epicanthal folds. We speculate that the advantages of this surgical procedure were the replacement of the original horizontal skin of the epicanthal folds with excess skin at the upper eyelid in the horizontal direction, inward rotation of the upper eyelid flap to fully supplement the insufficient vertical skin at the medial canthus, and removal of the redundant skin and orbicularis oculi muscle of the upper eyelid margin flap according to the skin relaxation and degree of flap rotation. As the curvature ratio of the rotating flap was significantly lower than 4:1, there was no tension during suturing, and a tension scar was not easily formed.

This study showed that the modified asymmetric inverse Z-plasty was more effective in hiding scars than Z-plasty, particularly in patients with moderate and severe epicanthal folds. The patients who underwent this technique were more satisfied. In Z-plasty, the epicanthal fold margin represented the central axis with equal-length arms and triangular flaps to reduce the horizontal epicanthal folds. However, the limited flap angle design provided insufficient vertical skin, causing high tension during suturing. Furthermore, the central axis of Z-plasty was at the epicanthal fold margin, and the postoperative incision margin was more evidently exposed, making it challenging to hide the scars. After Z-plasty, the lateral limb adjacent to the lower eyelid was not aligned with the natural skin lines of the eye and was positioned further away from the lower margin of the medial canthus. This misalignment increased the tension on the wound during eye movement, increasing the risk for scar formation. Modified asymmetric inverse Z-plasty was performed using the vertical projection point of the lacrimal caruncle as the new starting point of the medial canthus, with the preoperative design of the long-arm length guiding the design and adjustment of excess skin excision. The excess skin in the long and short arms was cut to form a curved flap, which was closely aligned with the physiological shape of the medial canthus. The horizontal point of the curved flap without tension was sutured to the starting point of the medial canthus to form a new point. The flap was rotated to transfer the excess horizontal skin vertically, compensating for the lack of longitudinal skin. After the two flaps were transposed using modified asymmetric inverse Z-plasty, the short-arm flap and its extended incision were located at the medial upper palpebral margin, making the scar almost hidden. Our modified surgical technique reconfigured the postoperative wound after Z-plasty by reducing the three limbs to two, specifically optimizing the third limb near the inner corner of the lower eyelid to minimize scar formation in the infraorbital region. Moreover, due to the appropriate removal of excess skin and the orbicularis oculi muscle during the extended eyelid margin incision, the upper and lower lip tissues formed thick and thin layers in the medial upper eyelid, respectively, favoring double eyelid formation. The modified asymmetric inverse Z-plasty was more effective for scar control and exhibited higher patient satisfaction than did Z-plasty, particularly for patients with moderate and severe epicanthal folds. However, as only female participants were involved in this analysis, the sex distribution of participants was uneven, which may introduce a bias and affect the results. Further randomized studies with larger sample sizes are required to confirm our findings.

Skin redraping technique has become a prevalent epicanthoplasty technique recently. It is preferred by plastic surgeons due to its straightforward surgical design, ease of execution, and consistently favorable therapeutic outcomes [22]. Skin redraping fully releases the subcutaneous tissues of the medial canthus and alleviates the skin tension caused by ligament traction. Similarly, it removes excess skin, enlarging the inner corner of the eye [23]. However, the lack of rotational flaps to complement the vertical skin results in the incomplete resolution of the vertical tension associated with the epicanthal folds. Furthermore, compared with the modified asymmetric inverse Z-plasty in this study, skin redraping causes a relatively longer lower eyelid wound, increasing the possibility of scar formation. Given that the incision under skin redraping is closer to the lower eyelid margin of the medial canthus, postoperative wound suturing and traction may alter the original physiological curvature of the medial canthal lower ring. This could somewhat compromise the aesthetic appearance of the patient’s eyes following surgery. In addition, epicanthoplasty performed in conjunction with double eyelid surgery is a well-established and common cosmetic procedure among Asians [24]. In terms of upper eyelid double eyelid surgery, our modified approach may facilitate integration with various double eyelid designs, enhancing aesthetic outcomes. Nevertheless, the aforementioned speculations require more rigorous clinical comparative experiments for further validation. Our subsequent research will likely prioritize this topic.

## Conclusion

Irrespective of the surgical method, “no scarring” cannot be guaranteed after epicanthoplasty. However, compared to Z-plasty, the modified asymmetric inverse Z-plasty demonstrated better scar hiding effects and higher patient satisfaction, particularly in patients with moderate and severe epicanthal folds. Hence, it can be considered an effective and safe treatment for epicanthal folds. This technique may influence the choice of the surgical approach for epicanthoplasty, offering an effective and safe option to minimize visible scarring. Further research should explore long-term outcomes and refine the technique for broader clinical use.
